# A decrease in functional microbiomes represented as *Faecalibacterium* affects immune homeostasis in long-term stable liver transplant patients

**DOI:** 10.1080/19490976.2022.2102885

**Published:** 2022-08-11

**Authors:** Soon Kyu Lee, JooYeon Jhun, Seung Yoon Lee, Sukjung Choi, Sun Shim Choi, Myeong Soo Park, Seon-Young Lee, Keun-Hyung Cho, A Ram Lee, Joseph Ahn, Ho Joong Choi, Young Kyoung You, Pil Soo Sung, Jeong Won Jang, Si Hyun Bae, Seung Kew Yoon, Mi-La Cho, Jong Young Choi

**Affiliations:** a Division of gastroenterology and hepatology, Department of Internal Medicine, Incheon St. Mary’s Hospital, College of Medicine, The Catholic University of Korea, Seoul, Republic of Korea; b The Rheumatism Research Center, Catholic Research Institute of Medical Science, College of Medicine, The Catholic University of Korea, Seoul, Republic of Korea; c Department of Biomedicine & Health Sciences, College of Medicine, The Catholic University of Korea, Seoul, Republic of Korea; d Division of Biomedical Convergence, College of Biomedical Science, Institute of Bioscience & Biotechnology, Kangwon National University, Chuncheon, Korea; e Research Center, BIFIDO Co., Ltd, Hongcheon, Korea; f Department of Surgery, Seoul St. Mary’s Hospital, College of Medicine, The Catholic University of Korea, Seoul, Republic of Korea; g Division of gastroenterology and hepatology, Department of Internal Medicine, Seoul St. Mary’s Hospital, College of Medicine, The Catholic University of Korea, Seoul, Republic of Korea; h Division of gastroenterology and hepatology, Department of Internal Medicine, Eunpyeong St. Mary’s Hospital, College of Medicine, The Catholic University of Korea, Seoul, Republic of Korea; i Department of Medical Lifescience, College of Medicine, The Catholic University of Korea, Seoul, Republic of Korea

**Keywords:** Liver transplantation, immunosuppressant, gut microbiome, gut dysbiosis, tolerance, regulatory T cells, *Faecalibacterium*, bacteroides

## Abstract

This study aimed to document the functional microbiome affecting immune homeostasis in long-term post-liver transplantation (LT) patients. We compared the frequency of regulatory T (Treg) and T helper 17 (Th17) cells in the blood and fecal microbiome of 27 LT patients to matched healthy controls (n = 20) using flow cytometry and 16S rRNA sequencing. Among the 27 LT patients, 22 patients ingested immunosuppressants (long-term post-LT group) and five were tolerant patients. The changes in Treg and Th17 cell proportion after treatment with distinct microbiomes in the long-term post-LT group were also examined *in vitro* . Changes in the identified functional microbiome and Treg cells in tolerant patients were evaluated. The mean time after LT of the included patients was 13.2 y. The gut microbiome of the long-term post-LT group showed lower alpha-diversity (*P* < .05) with distinct overall microbial composition (*P* = .001) compared to healthy controls. Among the 11 distinct bacterial genera in abundance, *Faecalibacterium* was the most decreased in the long-term post-LT group. The long-term post-LT group also demonstrated a decrease in Treg with an increase in Th17 cells, recovered by administration of *F. prausnitzii* and butyric acid in *in vitro* analysis. In tolerant patients, *Faecalibacterium* was marginally increased, coupled with an increase in Treg cells, compared to the long-term post-LT group. In conclusion, the long-term post-LT patients showed a decrease in functional microbiomes represented as *Faecalibacterium* . These findings provide potential biomarkers for assessing immune status and targets for improving immune homeostasis in LT patients.

**Abbreviations:**

LT, liver transplantation; HCC, hepatocellular carcinoma; IS, immunosuppressants; DC, dendritic cells; Treg, regulatory T; Th17, T helper 17; AST, aspartate transaminase; ALT, alanine transaminase; OUT, operational taxonomic unit; LEfSe, linear discriminant analysis effect size; LDA, linear discriminant analysis; IL, interleukin; TGF, transforming growth factor; GM-CSF, granulocyte-macrophage colony-stimulating factor; IFN, interferon; TNF-α, tumor necrosis factor-α; MIP-1α, macrophage inflammatory protein-1α; IP-10, interferon γ-induced protein; MCP-1, monocyte chemoattractant protein-1; ACR, acute cellular rejection; NF-κB, nuclear factor κB; PT INR, prothrombin time; QC, quality check; PBMC, peripheral blood mononuclear cells; PBS, phosphate-buffered saline; ELISA, enzyme-linked immunosorbent assay

## Introduction

Liver transplantation (LT) is the ultimate treatment for end-stage liver diseases or early hepatocellular carcinoma (HCC), improving hepatic function and treating HCC simultaneously.^1^ Advances in the development of immunosuppressants (IS) and post-LT care improved long-term survival after LT.^2^ With the unique immunological features in the human liver allografts, approximately 5%–20% of selected LT patients could achieve tolerance. In contrast, some patients still have a possibility of rejection even in the long term after LT.^3^ In mechanism, donor-derived dendritic cells (DCs), natural killer T cells, and CD4^+^ regulatory T (Treg) cells play important roles in successfully minimizing IS or tolerance after LT.^4,5^ Indeed, in our previous study, an increase in the Treg cells indicates the possibility of depreciation and tolerance during tapering IS.^6^ Although Treg cells play an important role in maintaining immune homeostasis, the effect of the gut microbiome on Treg cells in patients with long-term post-LT and tolerance remains unclear.

Gut microbiota modulates systemic immune functions along the gut–liver axis.^7^ In end-stage liver disease, gut dysbiosis is ubiquitous; it increases lipopolysaccharide and microbial metabolites, which induce inflammatory cytokines and modulate adaptive immune function with a decrease in Treg and an increase in T helper 17 (Th17) cells.^8–10^ In the early phase of post-LT, gut dysbiosis could persist and even get worse with a decrease in potentially beneficial genera, including *Faecalibacterium, Bifidobacterium* , and *Lactobacillus* .^11,12^ A higher *Proteobacteria* and lower *Firmicutes* , including *Faecalibacterium* , are even correlated with posttransplant cognitive impairment.^13^ This gut dysbiosis partially recovers within 12 to 24 months post-LT.^8^ However, whether the gut microbial balance is fully recovered in long-term post-LT patients remains unclear. Moreover, identifying functional microbiomes affecting immune homeostasis under the influence of long-term IS has not yet been evaluated.

Tacrolimus, one of the main IS after LT, has decreased gut diversity compared to other IS in patients with renal transplantation.^14^ Because most long-term post-LT patients are still taking a low-dose of IS, evaluating gut microbial balance and differences compared to healthy controls is needed to identify the functional microbiome affecting immune homeostasis by controlling Treg and Th17 cells. Moreover, whether gut microbiome in long-term post-LT patients is changed and recovered after achieving tolerance remains unclear. Therefore, the evaluation and identification of functional microbiomes along with their impacts on the immune system in long-term post-LT and tolerant patients may provide potential biomarkers reflecting the immune status of LT patients.

Herein, for the first time, we evaluated and compared the gut microbiome among long-term post-LT patients, healthy controls, and tolerant LT patients. Subsequently, we identified functional microbiomes affecting immune homeostasis, including Treg and Th17 cells, in long-term post-LT patients, and studied their effect on Treg and Th17 cells via *in vitro* analysis.

## Results

### Baseline characteristics of the entire population

The clinical and demographic parameters of 22 long-term post-LT patients and five tolerant patients included in the study are summarized in Table 1. The mean age was 63.0 ± 6.8 y, and 48.1% (n = 13) of patients were male. Living donor LT accounted for about 74% of the included patients, and hepatitis B virus-associated liver disease (n = 25, 92.6%) was the most common cause of LT. Of the 22 long-term post-LT patients, 17 patients (77.3%) ingested tacrolimus with a mean dose of 2.1 ± 0.9 mg per day. The other 5 patients (22.7%) ingested cyclosporine (mean dosage, 115.0 ± 33.5 mg/day). All 27 patients had a normal liver function, including aspartate transaminase (AST), alanine transaminase (ALT), albumin, and total bilirubin levels. The mean time after LT of 27 included patients was 13.2 ± 4.9 y without significant difference between the two groups. Twenty-five patients who had hepatitis B virus-associated liver disease before LT showed negative HBsAg with positive HBsAb after LT (Supplementary table 1). All those 25 patients ingested antiviral agents (n = 22, entecavir; n = 1, tenofovir; n = 2, lamivudine) and, of them, 21 patients also received hepatitis B immune globulin every 2–3 months. No patients experienced reactivation of hepatitis B during follow-up.

**Table 1.** Baseline characteristics of entire population.

Variables	Total (N = 27)	Long-term post-LT patients (n = 22)	Tolerant patients (n = 5)	P-value
Age, years	63.0 ± 6.8	62.3 ± 7.3	66.2 ± 2.4	0.053
Male sex (n,%)	13 (48.1%)	11 (50.0%)	2 (40.0%)	1.000
LDLT	20 (74.1%)	18 (81.8%)	2 (40.0%)	0.091
Cause of LT				
– LC/HCC/ALF	14 (52%)/11 (41%)/2 (7%)	11 (50%)/9 (41%)/2 (9%)	3 (60%)/2 (40%)/0 (0%)	1.000
– HBV/other	25 (92.6%)/2 (7.4%)	21 (95.5%)/1 (4.5%)	4 (80%)/1 (20%)	0.342
Type of IS				
– Tacrolimus/CsA	17 (77.3%)/5 (22.7%)	17 (77.3%)/5 (22.7%)	0/0	
Dose of IS (mg/day)				
– Tacrolimus/ CsA	2.1 ± 0.9/115.0 ± 33.5	2.1 ± 0.9/115.0 ± 33.5	0/0	
Level of IS (ng/mL)				
– Tacrolimus/ CsA	3.9 ± 1.7/101.3 ± 46.3	3.9 ± 1.7/101.3 ± 46.3	0/0	
AST (U/L)	23.0 ± 6.0	23.6 ± 6.1	20.6 ± 5.5	0.316
ALT (U/L)	23.5 ± 14.2	24.6 ± 15.5	18.8 ± 4.0	0.532
Total bilirubin (mg/dL)	0.7 ± 0.9	1.1 ± 0.7	0.6 ± 0.1	0.007
Albumin (g/dL)	4.4 ± 0.3	4.4 ± 0.3	4.4 ± 0.1	0.793
ALP (mg/dL)	67.1 ± 21.8	66.5 ± 21.2	69.4 ± 26.6	0.797
r-GTP (mg/dL)	50.3 ± 53.3	49.1 ± 50.7	56.0 ± 70.0	0.574
INR	1.0 ± 0.1	1.0 ± 0.0	1.0 ± 0.1	0.461
Platelet (x10^9^/L)	189.3 ± 42.8	182.5 ± 34.9	219.4 ± 64.2	0.081
Post-LT, years	13.2 ± 4.9	12.8 ± 4.6	15.1 ± 6.3	0.359

LT, liver transplantation; LDLT, living donor liver transplantation; LC, liver cirrhosis; HCC, hepatocellular carcinoma; ALF, acute liver failure; IS, immunosuppressive drugs; CsA, cyclosporine; AST, aspartate aminotransferase; ALT, alanine aminotransferase; ALP, alkaline phosphatase; r-GTP, gamma glutamyl transferase; INR, international normalized ratio

### Diversity and composition of the gut microbiome in long-term post-LT patients

**Figure 1.**
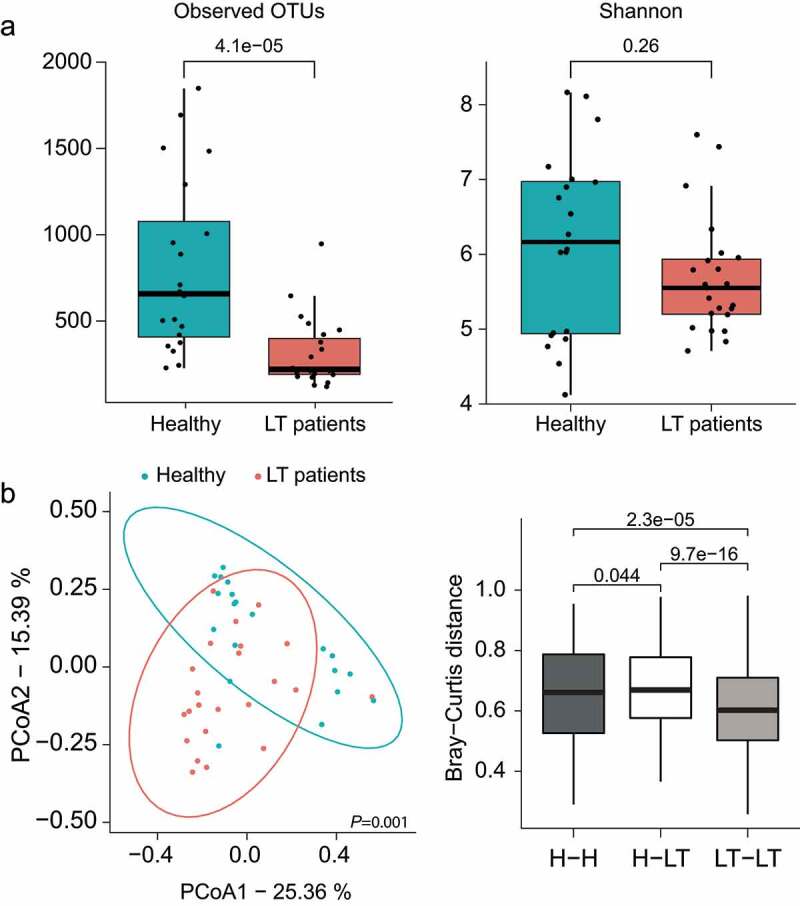
Comparisons of (a) alpha-diversity measured by observed OTUs and Shannon score and (b) beta-diversity using PCoA based on Bray-Curtis dissimilarity between long-term post-LT patients (n = 22) and healthy controls (n = 20). OTUs, operational taxonomic units; PCoA, principal coordinate analysis; LT, liver transplantation.

First, we evaluated the alpha diversity using observed operational taxonomic units (OTUs) and the Shannon diversity index. The long-term post-LT patients (n = 22) showed a decrease in intraindividual diversity compared to healthy controls (n = 20), as measured by the number of observed OTUs (*P* = 4.1e-05) and Shannon index (*P* = .26), which were statistically evaluated using the Mann–Whitney rank-sum test (Figure 1a).

We assessed and compared the overall diversity in fecal microbial composition between the long-term post-LT patients and healthy controls using principal coordinate analysis based on Bray-Curtis dissimilarity (beta diversity). Permutational multivariate analysis of variance showed a significant difference between long-term post-LT patients and healthy controls (R^2^=0.081, *P* = .001). Moreover, Bray–Curtis dissimilarity demonstrated that the microbial communities were significantly different between the two groups compared to each group (*P* < .05; Figure 1b).

### Differences in the abundant microbiome and immune homeostasis between long-term post-LT patients and healthy controls

**Figure 2.**
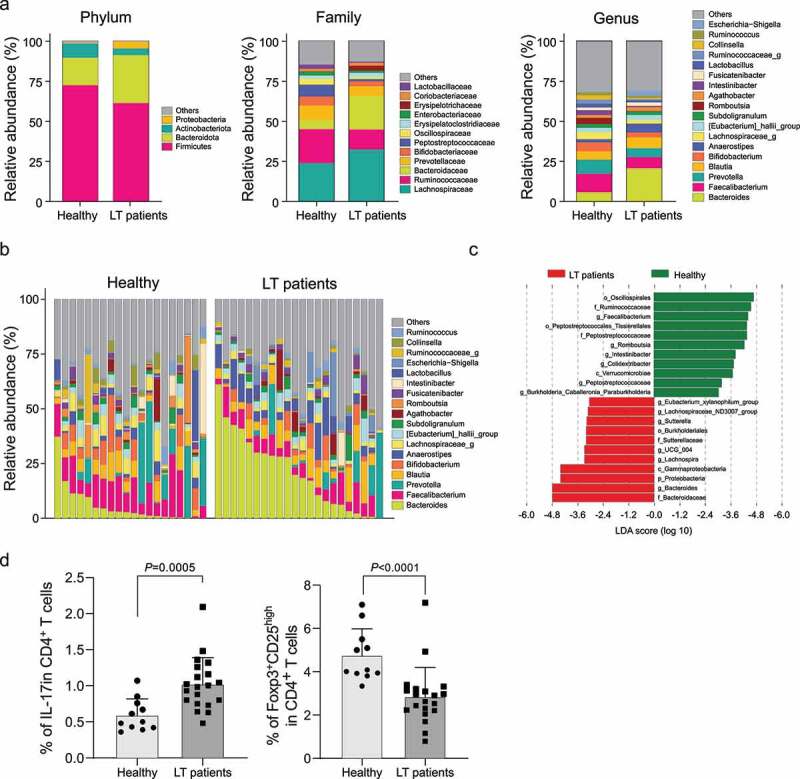
Comparisons of fecal microbial composition and the proportion of Treg and Th17 cells between long-term post-LT patients and healthy controls. (a, b) Relative proportion of bacteria at the phylum, family and genus level. (c) The LDA effect size analysis to identify significantly different microbiome in the relative abundance between long-term post-LT patients and healthy controls. (d) The proportion of CD4+ Treg and CD4+ Th17 cells in PBMC of long-term post-LT patients and healthy controls. Treg, regulatory T cells; Th17, T helper 17 cells; LT, liver transplantation; LDA, linear discriminant analysis.

Because of the different diversity in fecal microbial composition between long-term post-LT patients and healthy controls, we analyzed the linear discriminant analysis effect size (LEfSe) on the fecal microbiome to identify differences in the abundance microbiome between the two groups (Figure 2a-c). At the phylum level, *Firmicutes* and *Bacteroidetes* were dominant in both long-term post-LT patients and controls. Interestingly, *Proteobacteria* , including pathogenic species, were significantly increased in long-term post-LT patients. At the family level, significant differences were observed between the two groups with a decrease in *Ruminococcaceae* and *Peptostreptococcaceae* , and an increase in *Bacteroidaceae* in long-term post-LT patients.

There were also significant differences at the genus level with distinct relative abundances of 11 bacterial taxa between long-term post-LT patients and healthy controls (linear discriminant analysis [LDA] score >3.0, *P* < .05). Long-term post-LT patients showed a decreased abundance of *Faecalibacterium, Romboutsia, Intestinibacter, Colidextribacter, Peptostreptococcus* , and *Burkholderia* , and an increased abundance of bacteria including *Eubacterium, Lachnospira, Sutterella* , and *Bacteroides* . Notably, of the low-abundance bacteria in the long-term post-LT patients, the *Faecalibacterium* genus showed the strongest correlation. On the contrary, the *Bacteroides* genus demonstrated the strongest correlation in an increased abundance of bacteria in the long-term post-LT patients.

Along with these differences in the abundant microbiome, we also compared the population of Th17 and Treg cells between long-term post-LT patients and healthy controls. Intriguingly, the long-term post-LT patients demonstrated a decrease in the percentage of CD4^+^Treg cells (*P* < .01) with an increase in the proportion of Th17 cells (*P* < .01) compared to healthy controls (Figure 2d).

### Functional microbiomes represented as *Faecalibacterium* affect immune homeostasis in long-term post-LT patients

**Figure 3.**
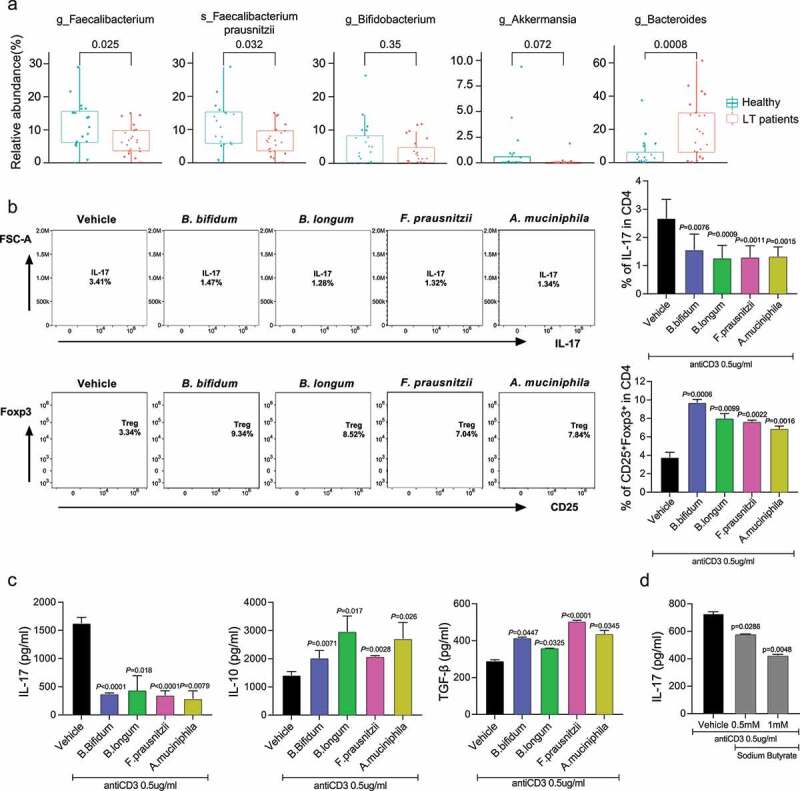
Comparisons of relative abundance of specific bacteria between long-term post-LT patients and healthy controls, and the change of Treg cells, IL-17, and IL-10 after treated with bacteria and butyrate. (a) Comparisons of relative abundance of the *Faecalibacterium, Bifidobacterium, Akkermansia, Bacteroides* genus and *F. prausnitzii* species between long-term post-LT patients and healthy controls. (b-d) CD4 + T cells from PBMC of long-term post-LT patients were cultured in the absence or presence of *B. bifidum, B. longum, F. prausnitzii, A. muciniphila* , and butyrate under the anti-CD3 stimulation condition for 3 d. The proportion of Treg cells and Th17 cells were analyzed by flow cytometry and the level of level of IL-17, IL-10 and TGF-β were analyzed by ELISA. LT, liver transplantation.

Considering the significant difference in the abundance of bacteria and immunological imbalance in the long-term post-LT patients, we next tried to identify the functional microbiomes affecting immune homeostasis in these patients. As shown in Figure 2d and Figure 3a, the *Faecalibacterium* genus and its species, *Faecalibacterium prausnitzii* , was significantly decreased (*P* = .025 and *P* = .0032, respectively). The *Bacteroides* genus was increased (*P* = .0008) in the long-term post-LT patients than in healthy controls, which were the most decreased and increased abundance of bacteria in the long-term post-LT patients, respectively. The *Bifidobacterium* genus and its species, *Bifidobacterium longum* , and *Bifidobacterium bifidum* , were marginally decreased in the long-term post-LT patients (Figure 3a and Supplementary Figure 1A). Moreover, the *Akkermansia* genus and its species, *Akkermansia muciniphila* , were considerably decreased and nearly undetectable in the long-term post-LT patients (Figure 3a and Supplementary Figure 1A).

To verify the immunomodulatory function of microbiomes, including *F. prausnitzii, B. longum, B. bifidum* , and *A. muciniphila* , in the long-term post-LT patients, we analyzed the differences in the proportion of Treg cells, Th17 cells and the levels of interleukin (IL)-17, IL-10 and transforming growth factor (TGF)-β in *in vitro* stimulated cells of the long-term post-LT patients after treatment with these bacteria. The population of Treg cells was significantly increased after treatment with *F. prausnitzii* (*P* = .0022), *B. longum* (*P* = .0099), *B. bifidum* (*P* = .0006), and *A. muciniphila* (*P* = .0016). Meanwhile, the population of Th17 cells was significantly decreased after treated with *F. prausnitzii, B. longum, B. bifidum* , and *A. muciniphila* (Figure 3b). Moreover, with the administration of *F. prausnitzii, B. longum, B. bifidum* , and *A. muciniphila* , the levels of IL-17, a pro-inflammatory cytokine, were profoundly decreased. In contrast, the levels of IL-10 and TGF-β, anti-inflammatory cytokines, were significantly increased (Figure 3c). As the *Faecalibacterium* genus is the major butyrate-producing bacteria, we also monitored the changes in the level of IL-17 after treatment with sodium butyrate to further validate the effect of *F. prausnitzii* and its metabolites on immune homeostasis. IL-17 level in *in vitro* stimulated cells of the long-term post-LT patients was also significantly reduced by the administration of sodium butyrate (Figure 3d).

### Comparison of gut microbiome including *Faecalibacterium* according to IS

**Figure 4.**
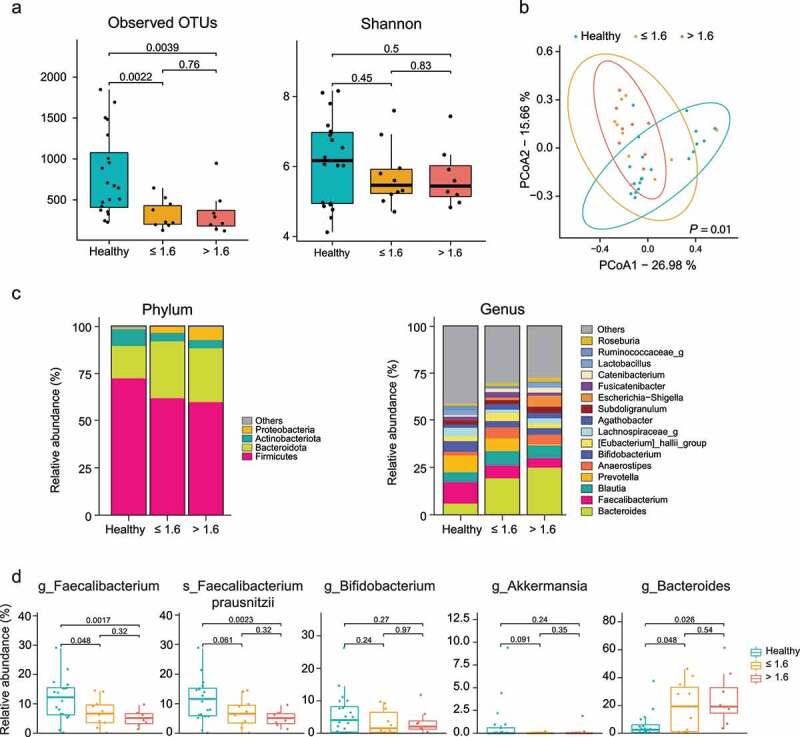
Comparisons of (a) alpha diversity measured by observed OTUs and Shannon score, (b) beta-diversity using PCoA based on Bray-Curtis dissimilarity, (c) microbial composition, and (d) relative abundance of specific bacteria between the higher, lower ratio and healthy groups. OTUs, operational taxonomic units; PCoA, principal coordinate analysis; the higher ratio group, the group with the ratio of tacrolimus level/dose > 1.6; the lower ratio group, the group with the ratio of tacrolimus level/dose ≤ 1.6.

Next, to evaluate the effect of IS on microbial composition including *Faecalibacterium* , we analyzed the differences in microbial composition and abundance of microbiome according to tacrolimus level/dose ratio. The patients treated with tacrolimus (n = 17) were divided into two groups based on the median value (1.6) of tacrolimus level/dose ratio. There were no significant differences in baseline characteristics between the two groups (Supplementary Table 2). The higher (>1.6) and lower (≤1.6) ratio groups showed similar alpha-diversity values for observed OTUs (*P* = .76) and Shannon index (*P* = .83; Figure 4a). Meanwhile, beta-diversity was significantly different between the two groups (*P* = .01; Figure 4b).

In the LEfSe analysis on the fecal microbiome, the *Proteobacteria* phylum and its genus *Escherichia/Shigella* were significantly increased in the higher ratio group compared to that in lower ratio group (Figure 4c). We next assessed the abundance of functional microbiomes, including *Faecalibacterium* , which was identified by comparing long-term post-LT patients and healthy controls. First, the higher ratio group showed decreased abundance of the *Faecalibacterium* genus and *F. prausnitzii* compared to the lower ratio group and healthy controls. The *Bifidobacterium* and *Akkermansia* genera and their species also demonstrated similar trends with a decrease in the higher ratio group. However, the *Bacteroides* genus increased in the higher ratio group, followed by the lower and healthy controls (Figure 4d, Supplementary Figure 1B). Considering these results, we could assume that the functional microbiomes represented as *Faecalibacterium* might be affected by IS.

### Changes in gut microbiome including *Faecalibacterium* after tolerance

**Figure 5.**
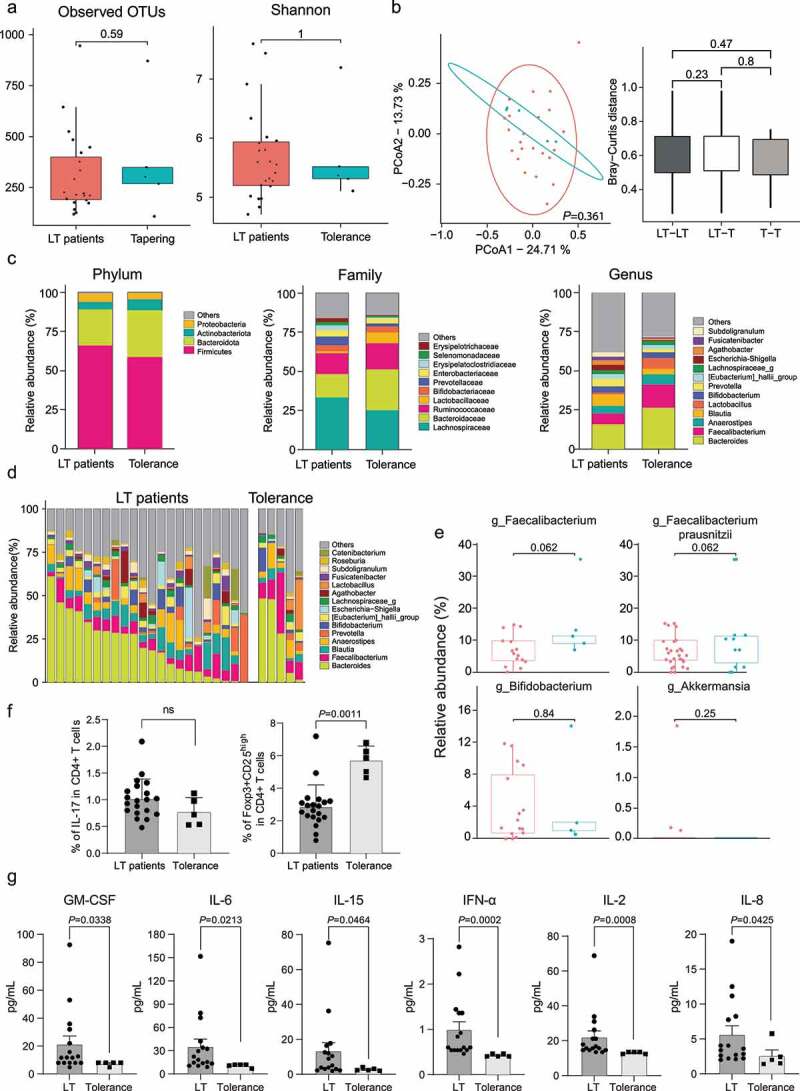
Comparisons of (a) alpha diversity measured by observed OTUs and Shannon score, (b) beta-diversity using PCoA based on Bray-Curtis dissimilarity, (c, d) microbial composition, (e) relative abundance of specific bacteria, and (f) the proportion of Treg and Th17 cells between long-term post-LT patients and tolerant patients. (g) Comparison of cytokine and chemokine expression in long-term post-LT patients and tolerant patients. Cytokines and chemokines were detected from plasma. OTUs, operational taxonomic units; PCoA, principal coordinate analysis; Treg, regulatory T cells; Th17, T helper 17 cells; LT, liver transplantation; GM-CFS, granulocyte-macrophage colony-stimulating factor; IL, interleukin; IFN, interferon.

Finally, we evaluated the microbial composition of tolerant patients (n = 5) and compared them with long-term post-LT patients (n = 22). The tolerant patients showed similar intraindividual diversity with long-term post-LT patients, as estimated by observed OTUs (*P* = .59) and Shannon index (*P* = 1.00) (Figure 5a). Beta-diversity was marginally different between the tolerant and long-term post-LT patients, as measured by permutational multivariate analysis of variance (*P* = .361) and Bray-Curtis dissimilarity (*P* = .23 and *P* = .80) (Figure 5b). Both groups demonstrated similar microbial composition at the phylum level, with *Firmicutes* being the most dominant bacteria, followed by *Bacteroidetes* and *Proteobacteria* (Figure 5c). At the genus level, *Faecalibacterium* tended to be increased in tolerant patients, whereas *Bacteroides* was slightly increased without significance (Figure 5c and 5d).

To evaluate whether the functional microbiome increases in tolerant patients, we compared the abundance of functional microbiomes represented as *Faecalibacterium* between tolerant and long-term post-LT patients. In tolerant patients, the abundance of the genus *Faecalibacterium* marginally increased (*P* = .053), and *Bifidobacterium* (*P* = .73) and *Akkermansia* (*P* = .35) genera were similar to those in long-term post-LT patients. Intriguingly, the *Akkermansia* genus did not recover and was nearly undetectable even after tolerance (Figure 5e). As *Faecalibacterium* was increased in tolerant patients, we next analyzed the population of Th17 and Treg cells in both groups to validate the impact of *Faecalibacterium* on immune homeostasis. The tolerant patients demonstrated a marginal decrease in Th17 cells with a significant increase in Treg cells (*P* < .001, figure 5f), which is similar to the results of the impact of *Faecalibacterium* on Th17 and Treg cells in *in vitro* analysis. Moreover, to examine the differences in cytokine and chemokine expression between LT patients and tolerant patients, multiplex assay using the ProcartaPlex Human 14-Plex Panel was performed (Figure 5g, Supplementary Figure 2). In the tolerant patients, the level of granulocyte-macrophage colony-stimulating factor (GM-CSF), IL-6, IL-15, interferon-α (IFN-α), IL-2, IL-1β, tumor necrosis factor-α (TNF-α), and IL-8 were significantly decreased compared to those in long-term post-LT patients. The level of macrophage inflammatory protein-1α (MIP-1α), interferon γ-induced protein (IP-10), monocyte chemoattractant protein-1 (MCP-1), IL-4, IL-12p70, and IL-23 were marginally diminished in tolerant patients.

## Discussion

This study is the first to evaluate the microbial composition and determine the functional microbiome affecting immune homeostasis in long-term post-LT patients. The long-term post-LT patients showed a decrease in intraindividual diversity and different fecal microbial compositions compared to healthy controls. Among the 11 distinct bacteria in abundance, *Faecalibacterium* and *Bacteroides* were the most decreased and increased bacteria in the long-term post-LT patients, respectively. Moreover, the long-term post-LT patients showed a decrease in Treg and an increase in Th17 cells, which could be recovered after administration of *Faecalibacterium* in *in vitro* analysis. The functional microbiome represented as *Faecalibacterium* showed a decreasing trend in patients with higher tacrolimus level/dose ratios, which was recovered in tolerant patients with an increase in Treg cells. Thus, these findings suggested that the long-term post-LT patients still have a decrease in functional microbiomes represented as *Faecalibacterium* , which led to an immune imbalance, proven by *in vitro* analysis and the functional microbiome in the tolerant patients.

Interestingly, our results suggested that the microbial composition of the long-term post-LT patients was still not fully recovered, showing a lower bacterial diversity and altered abundances compared to healthy controls. Although gut dysbiosis right after LT represented as a reduction in beneficial bacteria, *Faecalibacterium* , coupled with an increase in pathogenic genera, *Enterococcus* and *Bacteroides* ,^8,11,15^ is thought to be recovered partially in 12–24 months after LT,^8,11,12,16^ our study revealed that the microbial composition was not fully recovered even in long-term post-LT patients. In our study, gut dysbiosis in long-term post-LT patients was defined by increased abundance of five bacteria, including *Eubacterium* and *Bacteroides* , with decreased abundance of seven bacteria, including *Peptostreptococcus* and *Faecalibacterium* . Among these distinct bacteria in abundance, *Faecalibacterium* and *Bacteroides* were the most decreased and increased bacteria in the long-term post-LT patients, respectively. Intriguingly, these findings were in line with the microbial changes in animal model and patients with acute cellular rejection (ACR) after LT showing an increase in *Bacteroides* with a decrease in *Peptostreptococcus* and *Faecalibacterium* .^11,17,18^ Moreover, considering our results showing the persistence of gut dysbiosis coupled with a decrease in Treg cells and an increase in Th17 cells in long-term post-LT patients compared to healthy controls, the representative findings in LT patients with ACR,^6,19^ we could assume that a decrease in *Faecalibacterium* along with an increase in *Bacteroides* in the long-term post-LT patients might affect their immune homeostasis.

One of the key findings from our research was the identification of a functional microbiome, represented as *Faecalibacterium* (*F. prausnitzii*), by verifying their effects on immune homeostasis. In human cells stimulated *in vitro* from long-term post-LT patients, along with a decrease in IL-17 level, the population of Treg cells and the level of IL-10 and TGF-β were significantly increased after treatment with *F. prausnitzii* and sodium butyrate. These results might be explained by the anti-inflammatory properties of *Faecalibacterium* , including inhibition of nuclear factor κB (NF-κB), induction of Treg cells via interaction with CD103^+^DCs, and induction of high amounts of IL-10 in antigen-presenting cells.^20^ Moreover, our study revealed that butyrate, a metabolite produced by *F. prausnitzii* , also demonstrated similar immunomodulatory effects, including a decrease in IL-17 levels. Butyrate can reportedly inhibit the activation of macrophages and NF-κB, reduce T-cell activation, and promote induction of Treg cells.^21–23^ Indeed, reduced butyrate can lead to a decrease in the Treg/Th17 ratio, causing liver allograft rejection,^6,24^ whereas the restoration of butyrate can decrease apoptosis and mitigate graft-versus-host disease as per a previous study.^25^ In this regard, our finding that a decrease in Treg cells in the long-term post-LT patients might be attributable to a decreased abundance of *Faecalibacterium* and its metabolite, butyrate, provides insights into the potential of using functional microbiomes as biomarkers for assessing the immune status and as treatment targets for improving immune homeostasis in long-term post-LT patients.

It has not been well evaluated whether the abundance of functional microbiomes, including *Faecalibacterium* , differs according to IS in long-term post-LT patients. Although IS thought to affect the gut microbiome dose-dependently and only in the early phase of post-LT in previous studies,^16,26^ our study demonstrated trends toward a decrease in the abundance of the *Faecalibacterium* genus along with an increase in the abundance of the *Bacteroides* genus in the higher ratio group of long-term post-LT patients. In a rat model, the gut microbial composition has shown a close relationship with tacrolimus dosage, suggesting the importance of adjusting optimal IS dosage.^27^ Moreover, given that tacrolimus has a limitation of reducing Treg cells and decreasing overall gut microbial diversity and butyrate-producing species,^8,14,28^ the decreased abundance of *Faecalibacterium* in long-term post-LT patients might be attributable to tacrolimus, especially in the higher ratio group.

The immunomodulatory effects of functional microbiomes, including *Faecalibacterium* , were also verified in tolerant patients, who are considered immunologically stable without IS.^5,29^ The tolerant patients (n = 5) in our study had a long time after LT with a mean time of 15 y, a favorable factor (>10 y from LT) for inducing tolerance.^30^ Similar to our previous results demonstrating the importance of increasing Treg cells in tolerant patients during tapering IS,^6^ tolerant patients showed a higher proportion of Tregs than long-term post-LT patients taking IS. Moreover, the *Faecalibacterium* genus and *F. prausnitzii* were marginally increased in tolerant patients, supporting their immunomodulatory effects represented as inducing Treg cells.^20^ These results were also supported by the results of multiplex cytokine assay showing a decrease in pro-inflammatory cytokines including IL-2, IL-6, IL-8, IL-15, and GM-CSF in tolerant patients. To the best of our knowledge, this is the first study to demonstrate the change of functional microbiome along with Treg cells, Th17 cells, and cytokines in tolerant patients. Taken together, our results show that tolerant patients had higher abundances of *Faecalibacterium* , and a higher proportion of Tregs supports the notion that *Faecalibacterium* has potential properties as a biomarker and target for improving immune homeostasis and inducing tolerance.

Our study had several limitations. First, this study included a small number of patients from a single center. Second, we utilized 16S rRNA sequencing instead of metagenomic sequencing. However, this study was the first to evaluate the gut microbial composition and identify the functional microbiome, represented as *Faecalibacterium* , affecting immune homeostasis in long-term post-LT patients, which were verified by *in vitro* analysis and various patient groups including tolerant patients. Third, a marginal increase in *Faecalibacterium* in tolerant patients might be attributable to the small number of included tolerant patients. Considering the trends toward an increase in *Faecalibacterium* in tolerant patients, further research with more tolerant patients might confirm our results. Moreover, as host regional variation strongly affects the gut microbial composition,^31^ further multicenter studies including various regions are warranted to assess and verify our results in long-term post-LT patients. At last, as the authors used healthy controls as comparator group, microbial differences between LT patients and healthy controls might be due to LT as well as the long-term use of IS. However, other possible control groups such as non-LT patients on ISs might have other autoimmune diseases, which could generate another heterogeneous group. To evaluate the effect of IS, we also compared the microbial differences according to IS as well as between LT patients and tolerant patients, who withdraw IS. Finally, the authors demonstrated that functional microbiome represented as *Faecalibacterium* is still decreased in the long-term post-LT patients.

In conclusion, the long-term post-LT patients still showed a decrease in intraindividual diversity and differences in microbial composition, represented as a decrease in abundance in *Faecalibacterium* , leading to a decrease in Treg cells compared to Treg cells in healthy controls. Moreover, the immunomodulatory effects of *Faecalibacterium* in long-term post-LT patients were also proven by *in vitro* analysis and the findings in tolerant patients by augmenting Treg cells. Finally, these findings provide insight into the potential use of functional microbiomes, especially *Faecalibacterium* , as a biomarker for assessing immune status and a target for ameliorating immune homeostasis in long-term post-LT patients.

## Patients and Methods

### Patients

In this prospective cohort study, 27 LT patients were consecutively enrolled from an LT clinic at Seoul St. Mary’s Hospital between May 2019 and September 2020. All included LT patients (n = 27) underwent LT more than 5 y ago and had normal liver and renal functions. Among these patients, 22 patients ingested IS (the long-term post-LT group), and the other 5 had withdrawn IS successfully for more than 1 y (the tolerance group, n = 5).^29^ Patients who had a history of rejection, underlying autoimmune disease, other malignancy, or medical disease, including metabolic disease, were excluded from the study. Age-matched healthy controls (n = 20) without medical diseases, including metabolic and alcoholic diseases, were also recruited.

Patients’ clinical and laboratory findings at the time of fecal sampling were collected, including age, sex, LT date, type and cause of LT, type and drug level of IS, AST, ALT, albumin, total bilirubin, prothrombin time, and platelet count. This study was approved by the Institutional Review Board of the Catholic University of Korea (KC21TISI0113) and conducted in accordance with the Declaration of Helsinki. Informed consent was obtained from all the participants.

### Fecal DNA extraction, polymerase chain reaction (PCR) amplification and Sequencing

After collection in a plastic container, individual human fecal samples were immediately transported on ice to the research site and stored at −70°C within 12 h of arrival. Total DNA was extracted using the FastDNA® SPIN Kit for Soil (MP Biomedicals, USA), following the manufacturer’s instructions. PCR amplification was performed using fusion primers targeting the V3 to V4 regions of the 16S rRNA gene with the extracted DNA. For bacterial amplification, fusion primers 341 F (5′-AATGATACGGCGACCACCGAGATCTACAC-XXXXXXXX-TCGTCGGCAGCGTC-AGATGTGTATAAGAGACAG-CCTACGGGNGGCWGCAG-3′; underline sequence indicates the target region primer) and 805 R (5′- CAAGCAGAAGACGGCATACGAGAT-XXXXXXXX-GTCTCGTGGGCTCGG-AGATGTGTATAAGAGACAG-GACTACHVGGGTATCTAATCC-3′). The fusion primers were constructed in the following order: P5 (P7) graft binding, i5 (i7) index, NextEra consensus, sequencing adaptor, and target region sequence. The amplifications were carried out under the following conditions: initial denaturation at 95°C for 3 min, followed by 25 cycles of denaturation at 95°C for 30s, primer annealing at 55°C for 30s, and extension at 72°C for 30s, with a final elongation at 72°C for 5 min. The PCR product was confirmed using 1% agarose gel electrophoresis and visualized using a Gel Doc system (BioRad, Hercules, CA, USA). The amplified products were purified using CleanPCR (CleanNA). Equal concentrations of purified products were pooled together, and short fragments (non-target products) were removed with CleanPCR (CleanNA). The quality and product size were assessed on a Bioanalyzer 2100 (Agilent, Palo Alto, CA, USA) using a DNA 7500 chip. Mixed amplicons were pooled, and the sequencing was carried out at Chunlab, Inc. (Seoul, Korea), using the Illumina MiSeq Sequencing System (Illumina, USA) according to the manufacturer’s instructions.

### Data analysis pipeline

Processing raw reads started with quality check (QC) and filtering of low quality.^32^ After QC pass, paired-end sequence data were merged using fastq_mergepairs command of VSEARCH version 2.13.4 with default parameters.^33^ Primers were then trimmed with the alignment algorithm of Myers & Miller at a similarity cutoff of 0.8.^34^ Nonspecific amplicons that do not encode 16S rRNA were detected by nhmmer in HMMER software package ver. 3.2.1 with hmm profiles.^35^ Unique reads were extracted, and redundant reads were clustered with the unique reads by derep_fulllength command of VSEARCH.^33^ The EzBioCloud 16S rRNA database was used for a taxonomic assignment using the usearch_global command of VSEARCH followed by more precise pairwise alignment.^33,34,36^ Chimeric reads were filtered on reads with <97% similarity by reference-based chimeric detection using the UCHIME algorithm and the non-chimeric 16S rRNA database from EzBioCloud.^37^ After which, reads that were not identified at the species level (with <97% similarity) in the EzBioCloud database were compiled, and cluster_fast command2 was used to perform de novo clustering to generate additional OTUs. Finally, OTUs with single reads (singletons) were omitted from further analyses. The secondary analysis, which includes diversity calculation and biomarker discovery, was conducted using in-house programs of Chunlab, Inc. The alpha-diversity indices (ACE, Chao1,^38^ Jackknife,^39^ Shannon, NPShannon, Simpson, and Phylogenetic diversity), rarefaction curves, and rank abundance curves were estimated. Beta diversity distances were calculated using several algorithms (Jensen-Shannon, Bray-Curtis, Generalized UniFrac,^40^ Fast UniFrac).^41^ With functional profiles predicted by PICRUSt^42^ and MinPath^43^ algorithms, taxonomic biomarkers, and functional biomarkers were discovered by statistical comparison algorithms (LEfSe and Kruskal–Wallis H test).^44^ All the above mentioned analytics were performed in EzBioCloud 16S-based MTP, which is a ChunLab bioinformatics cloud platform. Raw 16S rRNA sequences were bioinformatically analyzed using QIIME 2 version 2019.4, as previously described.^45,46^

### Isolation and stimulation of peripheral blood mononuclear cells (PBMCs)

PBMCs were isolated from healthy controls and LT patients. Briefly, blood from healthy controls and LT patients was mixed with phosphate-buffered saline (PBS). The mixture was carefully layered onto 10 mL of Ficoll-Plaque Plus solution (GE Healthcare Life Sciences, Marlborough, MA, USA). Blood samples were centrifuged for 30 min at 1373xg and 20°C to separate the blood contents. After centrifugation, the layer containing PBMCs was collected. The cells were washed with PBS and cultured in RPMI medium containing 10% fetal bovine serum. PBMCs were incubated with plate-bound anti-CD3 (0.5 μg/mL) for 3 d; they were then subjected to IL-10 and IL-17 analysis by enzyme-linked immunosorbent assay (ELISA) and flow cytometry. A single suspension was prepared, and 5 × 10^5^ cells/well in 48 well flat bottom plates were cultured.

The PBMCs were incubated with plate-bound anti-CD3 (0.5 μg/mL) for 72 h in the presence of *B. bifidum* 4.5 × 10^6^ colony forming units (CFU)/mL, *B. longum* 1.0 × 10^6^ CFU/mL, F. *prausnitzii* 1.3 × 10^5^ CFU/mL, *A. muciniphila* 1.0 × 10^5^ CFU/mL and *sodium butyric acid* 0.5 mM, 1 mM. They were then subjected to IL-10, IL-17 and TGF-β and IL-17 analysis by ELISA and flow cytometry.

### Enzyme-linked immunosorbent assay

The supernatant was collected 3 d after *A. muciniphila, B. bifidum, B. longum, F. prausnitzii* , or *sodium butyric acid* or vehicle treatment with anti-CD3 (0.5㎍/mL). IL-10, IL-17, and TGF-β levels were assessed using sandwich ELISA (IL-10-DY217B, IL-17-DY317, and TGF-β-DY240 kit DuoSet ELISA; R&D Systems, Lille, France). The absorbance at 450 nm was measured using an ELISA microplate reader (Molecular Devices).

### Flow cytometry analysis

For intracellular cytokine staining for PBMC, prior to the intracellular staining, the cells were stimulated 50 ng/mL PMA (Sigma Aldrich, St. Louis, MO, USA) and 500 ng/mL ionomycin (Sigma-Aldrich) in the presence of GolgiStop (BD Bioscience, San Diego, CA, USA) for 4 h. After stimulation, cells were stained with fixable Viability Dye eFluor ^TM^780 (eBioscience, San Diego, CA, USA). After surface staining, cells were fixed and permeabilized with Cytofix/Cytoperm in accordance with the manufacturer’s instructions (BD Biosciences). Cytokine expression in human analyzed via intracellular staining with the following antibodies: PE anti-CD3: HIT3α (BD Bioscience), Alexa Fluor® 700 anti-CD4: RPA-T4 (BD Bioscience), FITC anti-CD8: HIT8α (BD Bioscience) and Alexa Fluor® 647 anti-IL-17: SCPL1362 (BD Bioscience). For intracellular Foxp3 staining, Buffer kit was used (eBioscience) after surface staining. After washing with Perm/Wash buffer, antibodies for Treg populations were stained with PE anti-CD3: HIT3α (BD Bioscience), Alexa Fluor® 700 anti-CD4: RPA-T4 (BD Bioscience), FITC anti-CD8: HIT8α (BD Bioscience), PerCP-Cy™5.5 anti-CD25: M-A251 (BD Bioscience) and Alexa Fluor® 647 anti-Foxp3: 236A/E7 (BD Bioscience). Stained cells were analyzed on a cytoFLEX Flow Cytometer (Beckman Coulter, Brea, CA, USA) (Supplementary Figure 3). At least of 5 × 10^4^ events of FSC/SSC lymphocyte gate of each experimental sample were acquired for adequate resolution. Flow cytometry data were analyzed using FlowJo (Tree Star, Ashland, OR, USA).

### Bacterial preparation

Commercial strains of *F. prausnitzii* ATCC 27768 and *A. muciniphila* ATCC BAA-835 were purchased from the American Type Culture Collection (Manassas, VA, USA). *F. prausnitzii* and *A. muciniphila* were cultured anaerobically for 24 h at 37°C in modified reinforced clostridial broth or brain heart infusion broth in Hungate tubes. *B. bifidum* and *B. longum* extracts (CTCBIO Korea Co., Ltd.) were resuspended in PBS. All the bacteria were heat-killed at 80°C for 30 min.

### Multiple bead based immunoassay

The multiplex cytokine assay, which is known as ProcartaPlex Human 14-Plex (PPX-14-MXPRMK7) Panel (Invitrogen^TM^, Thermo Fisher Scientific, Massachusetts, USA), was performed based on the Luminex® xMAP™ fluorescent bead-based technology (Luminex Corporation, 12,212 Technology Blvd, Austin, TX, 78727, USA). The 14 cytokines analyzed in the assay are as follows: GM-CSF, IFN-α, IL-1β, IL-2, IL-4, IL-6, IL-8, IL-12p70, IL-15, IL-23, IP-10, MCP-1, MIP-1α, and TNF-α. The plasma samples were collected and stored at −80°C until analysis. Sample aliquot has not been previously thawed before use in the multiplex cytokine and chemokine assay.

### Statistical analysis

The characteristics of patients are presented as the mean ± standard deviation or counts (percentage), as appropriate. Between-group comparisons for continuous variables were analyzed by Student’s *t* -test or analysis of variance for normally distributed variables, and Mann–Whitney *U* test or Kruskal–Wallis test for non-normally distributed variables. The Shapiro–Wilk test was performed to examine if a continuous variable follows a normal distribution. For categorical variables, the chi-square test or Fisher’s exact test were used. Statistical significance was set at 2-tailed, and *P* < .05. All statistical analyses were performed using R version 4.0.4 (http://cran.r-project.org) and GraphPad Prism 8.0 (GraphPad Software, Inc., San Diego, CA, USA)

## Supplementary Material

Supplemental Material

## Data Availability

The data that support the findings of this study are available in https://www.ncbi.nlm.nih.gov/bioproject/PRJNA863139, reference number PRJNA863139, and within the article and its supplementary materials
