# Supplementary material for: A decrease in functional microbiomes represented as Faecalibacterium affects immune homeostasis in long-term stable liver transplant patients
Source: Gut Microbes. 2022 Aug 11;14(1):2102885. doi: 10.1080/19490976.2022.2102885 (PMC9377238; doi:10.1080/19490976.2022.2102885)
Supplement: Supplemental Material [file KGMI_A_2102885_SM2776.docx]

**Supplementary Materials**

**Supplementary Figure 1.** Comparisons of relative abundance of *B. longum*, *B. bifidum*, and *A. muciniphila* between several groups: (A) healthy controls and long-term post-LT patients, (B) higher, lower ratio and healthy groups, and (C) long-term post-LT patients and tolerant patients. The higher ratio group, the group with the ratio of tacrolimus level/dose > 1.6; the lower ratio group, the group with the ratio of tacrolimus level/dose ≤ 1.6.


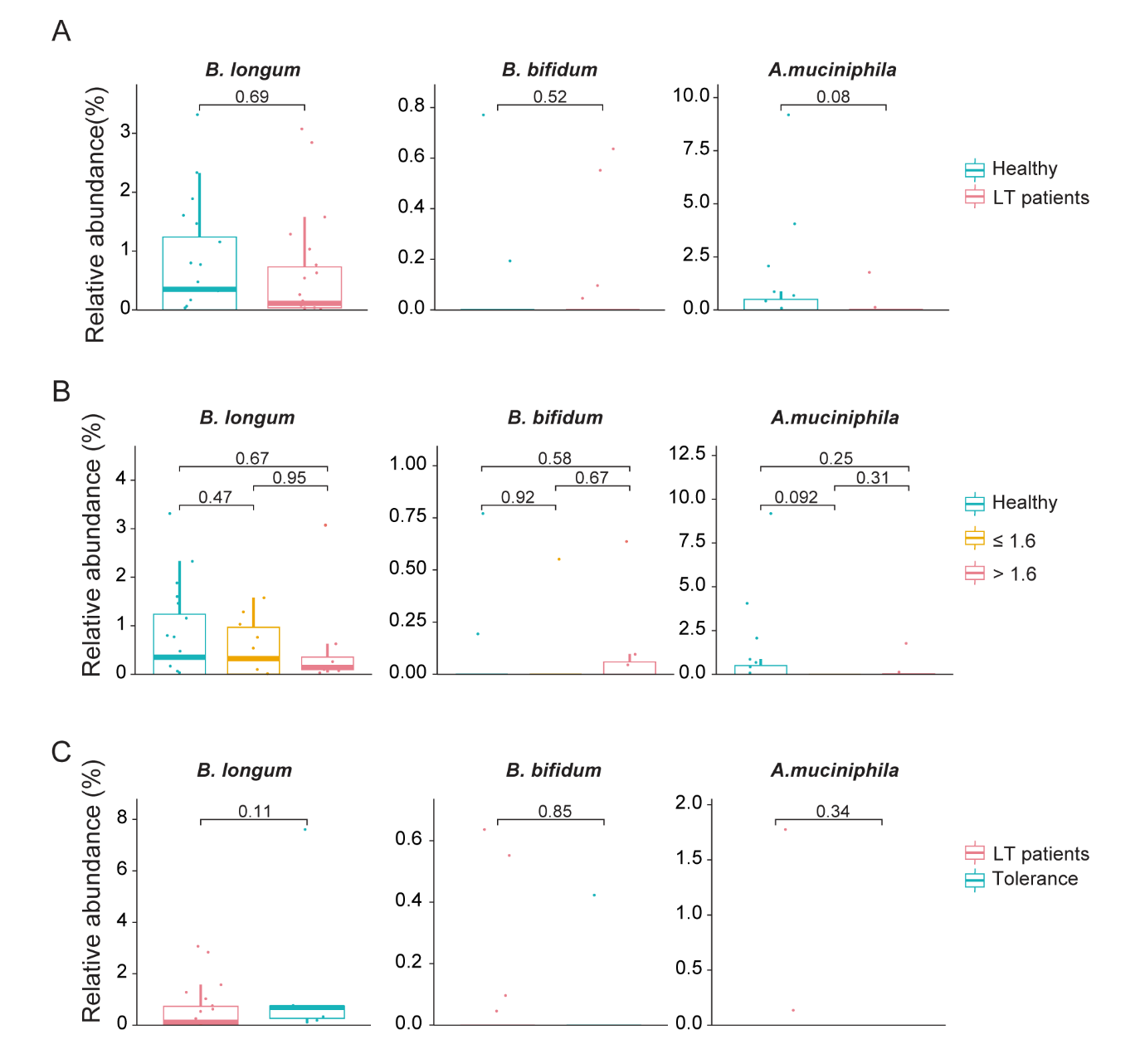


**Supplementary Figure 2.** Comparison of cytokine and chemokine expression in long-term post-LT patients and tolerant patients. Cytokine and chemokines were detected from plasma. MIP-α, macrophage inflammatory protein-1α; CCL, Chemokine ligand; IP-10, interferon γ-induced protein; CXCL, C-X-C Motif Chemokine Ligand; MCP-1, monocyte chemoattractant protein-1; IL, interleukin; TNF, tumor necrosis factor.

**
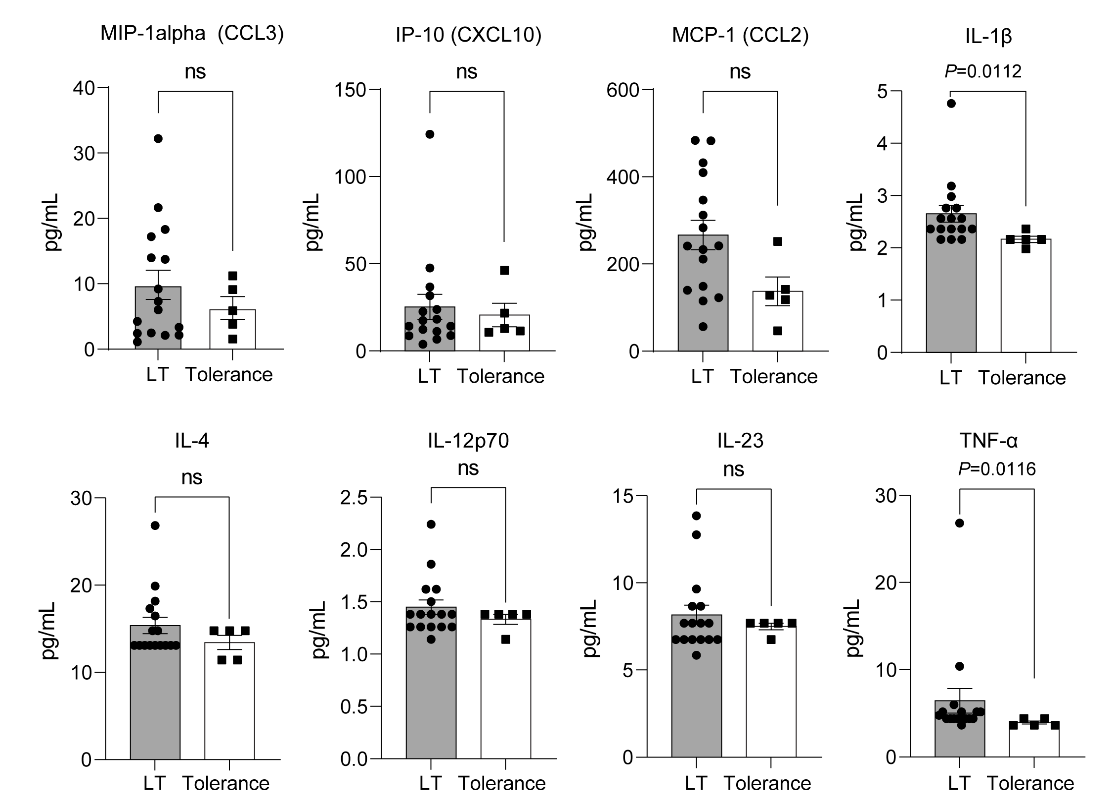
**

**Supplementary Figure 3.** Gating schematic for Th17 cells and Treg cells analysis

(A) Total cells were first gated on a Viable dye vs FSC to select live cells. Second, FSC-H vs FSC-A to select single cells amongst the pre-gated live cells. Third, CD3 vs FSC-A to select T-cells (CD3^+^) amongst the pre-gated live single cells. Forth, CD4 vs CD8 to select single-positive CD4+ T-cells (CD4^+^ CD8^-^) amongst the pre-gated live single T-cells. These were then further gated on IL-17+ population. (B) Total cells were first gated on a Viable dye vs FSC to select live cells. Second, FSC-H vs FSC-A to select single cells amongst the pre-gated live cells. Third, CD3 vs FSC-A to select T-cells (CD3^+^) amongst the pre-gated live single cells. Forth, CD4 vs CD8 to select single-positive CD4^+^ T-cells (CD4^+^ CD8^-^) amongst the pre-gated live single T-cells. Finally, FoxP3 vs CD25 to identify Tregs amongst the pre-gated CD4^+^ live single T-cells.


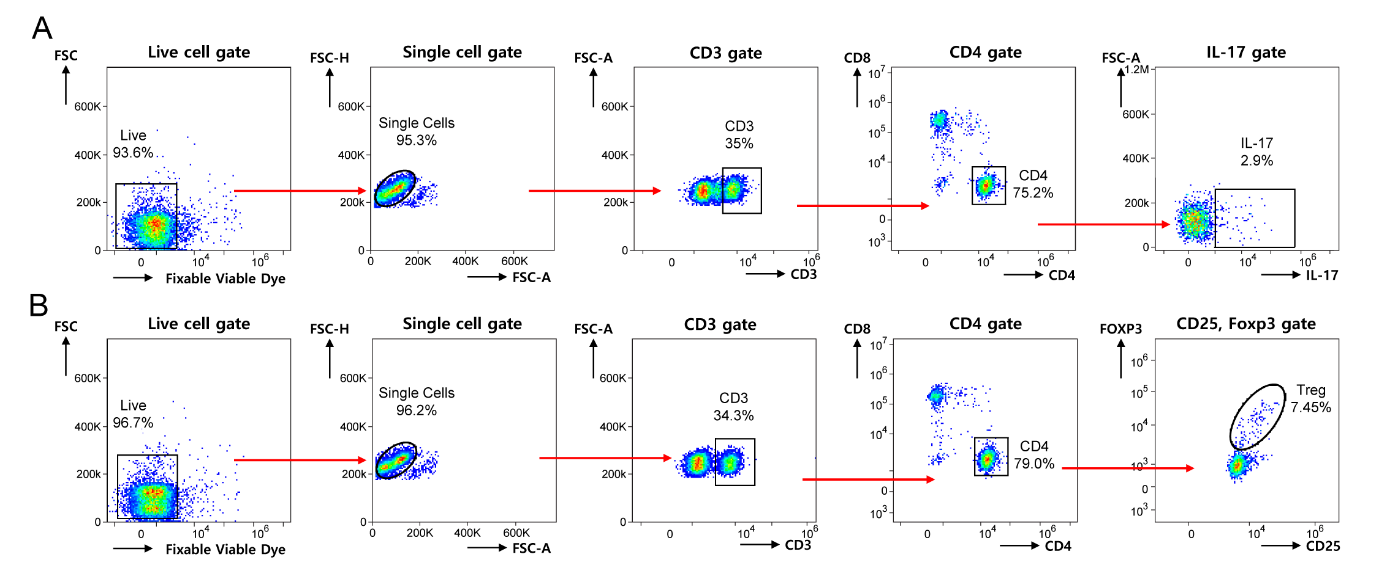


**Supplementary Table 1.** HBV status of long-term post-LT patients with hepatitis B before LT. HBV, hepatitis B virus; LT, liver transplantation

| **Variables** | **Total  (N=25)** | **Long-term post-LT patients (n=21)** | **Tolerant patients (n=4)** | ***P*-value** |
| --- | --- | --- | --- | --- |
| HBsAg positivity (n,%) | 25 (100%) | 21 (100%) | 4 (100%) | 1.000 |
| HBsAb positivity (n,%) | 24 (96.0%) | 20 (95.2%) | 1 (4.8%) | 1.000 |
| Usage of antiviral agent (n,%) | 25 (100%) | 21 (100%) | 4 (100%) | 1.000 |
| Type of antiviral agent |  |  |  | 1.000 |
| - ETV/TDF/LAM | 22 (88%)/1 (4%)/2 (8%) | 18 (86%)/1 (5%)/2 (10%) | 4 (100%)/0 (0%)/0 (0%) |  |
| Usage of HBIG (n,%) | 21 (84.0%) | 17 (81.0%) | 4 (100%) | 1.000 |
| LT, liver transplantation; ETV, entecavir; TDF, tenofovir; LAM, lamivudine; HBIG, hepatitis B immune globulin | | | | |

**Supplementary Table 2.** Baseline characteristics of long-term post-LT patients according to IS. LT, liver transplantation; IS, immunosuppressant

| **Variables** | **Tacrolimus group** | | ***P*-value** | |
| --- | --- | --- | --- | --- |
|  | **Level/dose ≤ 1.6 (n=9)** | **Level/dose > 1.6 (n=8)** |  |  |
| Age, years | 59.3 ± 7.9 | 62.1 ± 6.4 | | 0.438 |
| Male sex (n,%) | 4 (44.4%) | 4 (50.0%) | | 1.000 |
| LDLT | 7 (78.8%) | 7 (87.5%) | | 1.000 |
| Cause of LT |  |  | |  |
| - LC/HCC/ALF | 3(33%)/5(56%)/1(11%) | 5(62%)/2(25%)/1(13%) | | 0.536 |
| - HBV/other | 9 (100%)/ 0 (0%) | 5 (62.5%)/ 3 (37.5%) | | 0.082 |
| IS |  |  | |  |
| - Tacrolimus | 9 (100%) | 6 (100%) | | 1.000 |
| Dose of IS |  |  | |  |
| - Tacrolimus (mg/day) | 2.6 ± 0.8 | 1.6 ± 0.7 | | 0.196 |
| Level of IS |  |  | |  |
| - Tacrolimus (ng/mL) | 3.5 ± 1.2 | 4.3 ± 1.2 | | 0.321 |
| AST (U/L) | 22.0 ± 4.2 | 25.0 ± 8.6 | | 0.365 |
| ALT (U/L) | 22.2 ± 13.2 | 30.1 ± 21.1 | | 0.228 |
| Total bilirubin (mg/dL) | 0.9 ± 0.3 | 1.0 ± 0.2 | | 0.446 |
| Albumin (g/dL) | 4.4 ± 0.2 | 4.5 ± 0.4 | | 0.743 |
| ALP (mg/dL) | 59.3 ± 8.5 | 70.1 ± 13.9 | | 0.069 |
| r-GTP (mg/dL) | 42.1 ± 53.4 | 39.8 ± 28.1 | | 0.500 |
| INR | 1.0 ± 0.0 | 1.1 ± 0.0 | | 0.144 |
| Platelet (x10^9^/L) | 179.2 ± 28.8 | 183.0 ± 49.7 | | 0.848 |
| Post-LT, years | 11.0 ± 4.4 | 13.5 ± 4.6 | | 0.268 |
| LDLT, living donor liver transplantation; LT, liver transplantation; LC, liver cirrhosis; HCC, hepatocellular carcinoma; ALF, acute liver failure; AST, aspartate aminotransferase; ALT, alanine aminotransferase; ALP, alkaline phosphatase; r-GTP, gamma glutamyl transferase; INR, international normalized ratio | | | | |
